# Novel Functional MAR Elements of Double Minute Chromosomes in Human Ovarian Cells Capable of Enhancing Gene Expression

**DOI:** 10.1371/journal.pone.0030419

**Published:** 2012-02-03

**Authors:** Yan Jin, Zheng Liu, Wei Cao, Xinying Ma, Yihui Fan, Yang Yu, Jing Bai, Feng Chen, Jesusa Rosales, Ki-Young Lee, Songbin Fu

**Affiliations:** 1 Laboratory of Medical Genetics, Harbin Medical University, Harbin, China; 2 Department of Biochemistry and Molecular Biology, University of Calgary, Calgary, Canada; 3 Department of Cell Biology and Anatomy, University of Calgary, Calgary, Canada; The University of Hong Kong, China

## Abstract

Double minute chromosomes or double minutes (DMs) are cytogenetic hallmarks of extrachromosomal genomic amplification and play a critical role in tumorigenesis. Amplified copies of oncogenes in DMs have been associated with increased growth and survival of cancer cells but DNA sequences in DMs which are mostly non-coding remain to be characterized. Following sequencing and bioinformatics analyses, we have found 5 novel matrix attachment regions (MARs) in a 682 kb DM in the human ovarian cancer cell line, UACC-1598. By electrophoretic mobility shift assay (EMSA), we determined that all 5 MARs interact with the nuclear matrix in vitro. Furthermore, qPCR analysis revealed that these MARs associate with the nuclear matrix in vivo, indicating that they are functional. Transfection of MARs constructs into human embryonic kidney 293T cells showed significant enhancement of gene expression as measured by luciferase assay, suggesting that the identified MARS, particularly MARs 1 to 4, regulate their target genes in vivo and are potentially involved in DM-mediated oncogene activation.

## Introduction

Double minute chromosomes, also known as double minutes (DMs) are extrachromosomal elements (EEs) that are considered cytogenetic hallmarks of gene amplification [1]–[3]. The existence of DMs was initially observed in human colon carcinoma cells [4] but have now been shown in a variety of human tumors, including breast, lung, ovary, colon, and neuroblastoma [5]. DMs are small, generally acentric, atelomeric and autonomously replicating chromatin bodies. They are considered to be one of the principal genetic structures on which specific oncogenes are located [6]. It is believed that DM-mediated oncogene amplification or overexpression contributes to their oncogenic role [7]–[9]. Amplified DM sequences range in size from a few hundred kilobases to megabases. It is recognized that most of the amplified DM sequences are non-coding, but why cancer cells possess considerable amounts of extrachromosomal DNA requires further investigation.

Non-coding DNA is DNA sequence that does not encode a protein. More than 98% of the human genome is non-coding DNA, including most sequences within introns and intergenic DNA [10]. Although the sequence of the human genome has now been completed, the organization and function of non-coding DNA remains to be characterized. In the nucleus, high order chromatin structure is maintained by DNA-nuclear matrix interactions. DNA sequences that bind preferentially to the nuclear matrix are designated as matrix attachment regions (MARs) or scaffold associated regions (SARs). MARs/SARs, which are more often located in non-coding regions of DNA, are about 200 bp in length, AT-rich, and contain topoisomerase II consensus sequences and other AT-rich sequence motifs [11], [12]. They can activate gene expression, determine which class of genes to transcribe and have a strong effect on the level of transgene expression [13], [14]. Of the MAR elements reported, many do not display extensive sequence homology, but they appear to be functionally conserved, since animal MARs can bind to plant nuclear scaffolds and vice versa [15].

The human ovarian cancer cells, UACC-1598, stably harbors DMs. One of the DMs that we recently identified and sequenced was a 682 kb DM (NCBI Sequence Read Archive (SRA), Accession ID:SRA037306.1). Interestingly, this DM contains the oncogenes, MYCN and EIF5A2 [16]. Amplified copies of oncogenes in DMs have been associated with increased growth and survival of cancer cells but DNA sequences in DMs which are mostly non-coding remain to be characterized. It would be interesting to know whether certain MAR elements play a role in DM-mediated oncogene activation. In this report, bioinformatics analysis showed that the 682 kb DM harbors 5 matrix attachment regions (MARs). These MARs bind to the nuclear matrix of human ovarian cancer cells *in vitro* and *in vivo*, indicating that they are functional. They also enhance gene expression when inserted into the luciferase promoter region and transfected into 293T embryonic kidney cells. Together, our results suggest that the 682 kb DM MARs may play important roles in oncogene activation.

## Materials and Methods

### Cells and Cell Culture

The human ovarian cancer cell line UACC-1598, which spontaneously forms DMs, was kindly provided by Dr. XY Guan (The University of Hong Kong). The UACC-1598 cells were maintained in RPMI-1640 medium supplemented with 10% fetal bovine serum (GIBCO, Carlsbad, CA). HEK 293T cell line was purchased from the American Type Culture Collection (Manassas,VA) and was cultured in DMEM medium supplemented with 10% fetal bovine serum (GIBCO, Carlsbad, CA). Normal ovarian tissues were obtained from uterine cervix cancer patients at the Third Affiliated Hospitals of Harbin Medical University (Harbin, China).

### RT-PCR analysis

Total RNA was isolated using Trizol (Invitrogen), following the manufacturer's protocol. cDNA synthesis was performed using the First-Strand cDNA Synthesis Kit (Promega). β-actin was used as the internal PCR control. The PCR products were subjected to electrophoresis on a 1.5% agarose gel, and the bands were visualized using a gel documentation system (Alpha Innotech.).

### Western blot analysis

The cells and normal tissues were homogenized in RIPA buffer. Samples were then sonicated, vortexed and centrifuged at 12,000×g for 10 min at 4°C. The supernatants were collected and separated by SDS-polyacrylamide gels, blotted onto membranes and incubated with the primary antibody (MYCN from GeneTex, US; EIF5A2 from Protein Tech, Chicago; Histone H1, Santa Cruz; GAPDH from Santa Cruz Biotech) overnight at room temperature. Immunoreactive protein bands were detected using the ECL detection system (New England Biolab).

### Identification of MARs

MARs were identified using MAR-Wiz and MARFinder softwares (http://genomecluster.secs.oakland.edu) [17].

### Nuclei preparation

Nuclei was prepared as described by Cockerill et al [18]. Briefly, cells were harvested and washed once with phosphate-buffered saline (PBS), resuspended in RSB (10 mM Tris-HCl, pH 7.4; 10 mM NaCl; 3 mM MgCl; 0.5 mM PMSF), placed on ice for 10 min, and homogenized using a KONTES homogenizer. Nuclei were washed twice with RSB plus 0.25 M sucrose, resuspended in RSB plus 2 M sucrose, and centrifuged through a cushion of RSB plus 2 M sucrose at 14,000 rpm for 30 min. Isolated nuclei were washed once in RSB plus 0.25 M sucrose by centrifugation at 750×g for 10 min. Nuclei were resuspended in RSB plus 0.25 M sucrose, and after adding an equal volume of glycerol, were stored at −20°C.

### Isolation of nuclear matrix

Nuclei (∼1 mg/ml) in RSB containing 0.25 M sucrose and 1 mM CaCl_2_ were incubated with 100 µg/ml DNAase I (Fermentas, CA) for 2 hr at 23°C. After centrifugation at 750×g for 10 min at 4°C, pellets were resuspended in RSB containing 0.25 M sucrose, and an equal volume of cold 20 mM Tris buffer (pH 7.4) containing 4 M NaCl and 20 mM EDTA was added. After 10 min at 0°C, the suspension was centrifuged at 1,500×g for 15 min at 4°C, and pellets were extracted twice by resuspension in a cold solution of 10 mM Tris-HCl (pH 7.4) containing 2 M NaCl, 10 mM EDTA, 0.5 mM PMSF and 0.25 mg/ml BSA, and centrifugation at 4500×g for 15 min at 4°C. The resulting nuclear matrices were washed with cold RSB containing 0.25 M sucrose and 0.25 mg/ml BSA, resuspended in the same buffer, added an equal volume of glycerol, and stored at −20°C.

### EMSA analysis

DNA binding was analyzed by electrophoretic mobility shift assay (EMSA). Full-length DNA fragments of MARs amplified by PCR were incubated with isolated nuclear matrix at room temperature for 20 min. MARs-nuclear matrix interaction was analyzed by agarose gel electrophoresis and visualized under UV illumination using the Alpha Inotech Imaging System (Alpha Inotech Corporation, San Leandro, CA, USA).

### Isolation of nuclear matrix-associated DNA

Nuclei isolation and extraction of histones using lithium diiodosalicylate (LIS) were performed as described by Mirkovitch [10]. Ten units of nuclei (OD at 260 nm) in 100 µl of isolation buffer [5 mM Hepes pH 7.4, 0.25 mM spermidine, 2 mM KCl, 0.1% digitonin, 25 mM 3–5-diiodosalicylic acid lithium salt (Sigma)] were heated at 37°C for 20 min. Seven ml of low-salt extraction buffer [3.75 mM Tris-HCl, 0.05 mM spermine, 0.125 mM spermidine, 20 mM KCl, 1% (v/v) thiodyglycol, 0.1% digitonin] was then slowly added to the histone-free nuclei at room temperature. After 5 min, the histone-depleted nuclei were recovered by centrifugation at 2,400×g for 20 min at room temperature. The pellet was washed four times in 8 ml digestion buffer (20 mM Tris/HCl, pH 7.4, 0.05 mM spermine, 0.125 mM spermidine, 20 mM KCl, 70 mM NaCl, 10 mM MgCl_2_, 0.1% digitonin, 100,000 IU/ml Trasylol, and 0.1 mM PMSF). Restriction enzymes (EcoR1, HindIII, Xba1; New England Biolabs) were then added at 1000 U/ml and digestion was allowed to proceed for 3 hr at 37°C in a shaking water bath. The digested histone-depleted nuclei was centrifuged at 2,400×g for 10 min at 4°C to separate the solubilized DNA (S) from the nuclear scaffold-bound DNA (P) which was recovered and purified using QIAamp DNA Mini Kit-250 (QIAGEN) then resuspended in deionized water. DNAs were then analyzed by qPCR. As templates, P and S DNA were used. Genomic DNA (1598) isolated from UACC 1598 cells was used as control. The primers used for qPCR amplification are shown in Table S1.

### Vector construction

The identified MARs were amplified by PCR using the primers and annealing temperatures described in Table S2. Purified PCR products were digested with Kpn 1, Mlu 1, and Bgl II, and cloned into pGL3-promoter vector (Promega, Madison, WI).

### Transfection and luciferase assay

293T cells were transiently transfected with the prepared constructs (0.2 µg each) using the polyethylenimine (PEI) reagent (Polysciences, USA) according to the manufacturer's instructions. Cells transfected with the vector itself (0.2 µg) served as control. After 48 hours, cells were harvested and lysed with 1×lysis buffer (Promega, Madison, WI). Lysates were used to mesaure luciferase activity using the Dual-Luciferase Assay Kit (Promega, Madison, WI).

## Results

**Figure 1:**
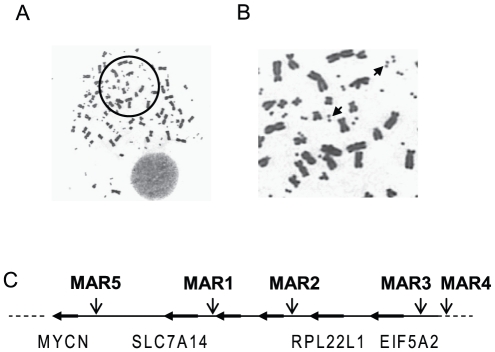
Sequencing of a 682 kb DM in UACC-1598 human ovarian cells revealed 5 putative MARs. A. Metaphase spread of UACC-1598 cells stained with DAPI (magnification, 200×). B. Magnified view of the circled area in (A). Arrows are directed at DMs. C. A schematic diagram of the 682 kb DM sequence in UACC-1598 cells shows 5 putative MARs using the MARFinder. The predicted MARs at 3q26 are located at base pairs 170327921–170328321 (MAR1), 170513721–170514421 (MAR2), 170633821–170634121 (MAR3) and 170634321–170634821 (MAR4). Another MAR is located on 2p24 at base pairs 15991750–15992550 (MAR5).

The 682 kb DM sequence in UACC-1598 human ovarian cells has 5 putative MARs. DMs are extrachromosomal elements that play a pivotal role in tumorigenesis. Analyses of metaphase spreads of UACC-1598 human ovarian cancer cells revealed numerous DMs (Figure 1, A and B). Recently, we cloned and sequenced a 682 kb DM from these cells and the data was submited to NCBI Sequence Read Archive (Accession ID:SRA037306.1), in collaboration with Ensembl, archives short read data from next-generation sequencing technologies (e.g. 454 Life Sciences [Roche], Illumina, ABI SOLiD, Helicos). While 97% of the sequence is non-coding, we identified five putative MAR elements in the sequence (Figure 1C) using the MAR-Wiz and MARFinder softwares as indicated in the Materials and Methods section.

### MARs 1 to 5 interact with the nuclear matrix in vitro

**Figure 2:**
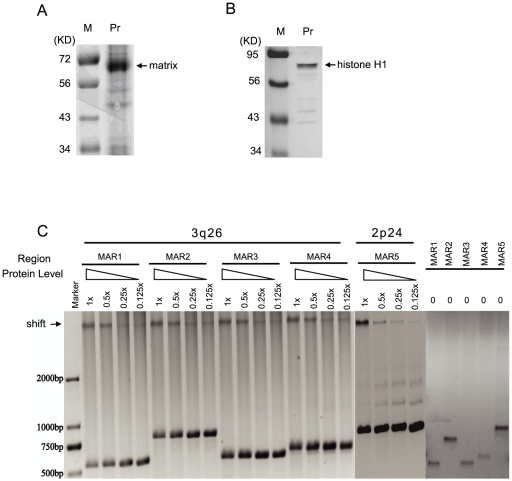
All identified MARs interact with the nuclear matrix in vitro. A. SDS-PAGE of the nuclear matrix purified from UACC-1598 cells. B. Western blot for nuclear matrix using histone H1 antibody. C. MARs 1 to 5 interact with the nuclear matrix in vitro. EMSA was performed as described in Materials and Methods using 100 ng DNA and serially diluted nuclear matrix.

To investigate whether the putative MARs 1 to 5 are functional, we performed *in vitro* EMSA assay to examine their ability to interact with the nuclear matrix. PCR amplified MARs 1 to 5 were incubated with nuclear matrix purified from UACC-1598 cells (Figure 2, A and B) and interaction between DNA and protein were assessed by mobility shift. As shown in Figure 2C, MARs 1 to 5 all interacted with the nuclear matrix, and upon dilution of the latter, a corresponding decrease in band shift (indicated by arrow) and increase in free MARs were observed. These results indicate that all five identified MARs bind to the nuclear matrix in a dose-dependent manner.

### MARs 1 to 5 interact with the nuclear matrix in vivo

**Figure 3:**
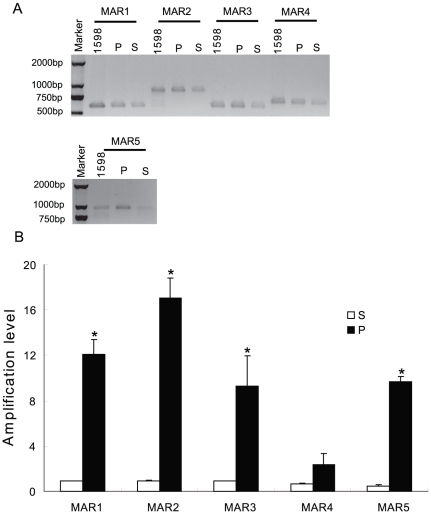
MARs 1 to 5 interact with the nuclear matrix in vivo. A. Matrix-associated (P) and solubilized (S) DNAs, isolated as described in Materials and Methods, were used as templates to amplify MARs 1 to 5 using primers listed in Table S1. Genomic DNA (1598) isolated from UACC 1598 cells was used as control. B. Densitometric analysis of qPCR band intensities is shown as column diagram. Asterisk indicates significance at p<0.05.

Next, we investigated whether the identified MARs 1 to 5 bind to the nuclear matrix *in vivo*. The matrix-associated DNA (P) was separated from the soluble DNA (S) of UACC-1598 human ovarian cells. The P and S fractions were used as templates to amplify MARs 1 to 5 by qPCR using primers listed in Table S1. As shown in Figure 3A and B, MARs 1 to 5 were clearly detectable in the P fraction, indicating that these MARs are associated with the nuclear matrix, and confirming that they are indeed functional MARS *in vivo*.

### MARs 1 to 5 differentially enhance gene expression

**Figure 4:**
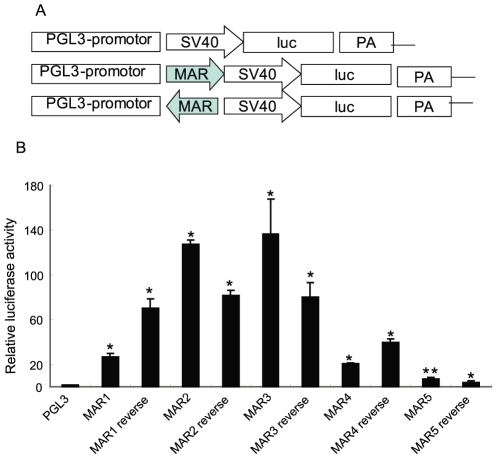
Differential enhancement of gene expression by MARs 1 to 5. A. Schematic diagram of the pGL3-MAR-luciferase reporter constructs in the 5′ to 3′ and 3′ to 5′ direction. B. Luciferase activity of 293T cell lysates after transfection with the constructs for 48 hours. Data represent the mean ± SD of three independent experiments. *, P<0.05; **, P<0.01.

**Figure 5:**
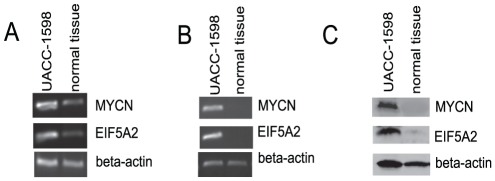
Upregulated detection of MYCN and EIF5A2 in UACC-1598. DNA amplification level of MYCN and EIF5A2 were evaluated by PCR(A). Expression of MYCN and EIF5A2 in UACC-1598 cells were determined by RT-PCR (B) and western blot analysis (C). Cells isolated from normal ovarian tissues were used as control. Primers for MYCN, EIF5A2 and β-actin are listed in Table S3.

MARs in DMs play important roles in the regulation of target genes. To test the possibility that our identified MARs can stimulate gene expression, we generated pGL3-MAR-luciferase reporter constructs (in both 5′ to 3′ and 3′ to 5′ direction) as shown in Figure 4A. After transfection of the constructs into 293T cells for 48 hours, cell lysates were measured for luciferase activity. As shown in Figure 4B, cells transfected with vector containing MARs 1 to 4, in either direction, showed significantly luciferase activity, with MARs 2 and 3 showing the highest activity. On the other hand, the vector containing MAR5 showed only modest luciferase activity. These data suggest that the identified MARs 1 to 4 may regulate a target gene(s) *in viv*o. Indeed, in UACC-1598 cells, we found upregulated expression of the MYCN and EIF5A2 genes which are localized close to these MARs (Figure 5).

## Discussion

The existence of DMs in maliganancies have been correlated with poor prognosis and outcome [5], [17], [19]–[21]. DMs have been found in a broad spectrum of human neoplasias with the highest frequency in neuroblastoma [5]. Several genes such as MYC, MYCN, DHFR, MDM2 and EGFR have been determined to be amplified on DMs [18], [22]–[25] but little is known about the precise structure and organization of DMs, and the mechanism by which DM is formed. In UACC-1598 human ovarian cells, we have identified a 682 kb DM with 97% non-coding DNA that consists of 5 functional MARs.

MARs are DNA sequences that can bind to the nuclear matrix in eukaryotes. They play critical roles in maintaining high order chromatin structure, determining the origin of DNA replication, and regulating gene expression. MARs have been shown to augment gene expression to varying extents in different systems through the modification of the chromatin structure [26], [27]. The different abilities of MARs in enhancing gene expression is due in part to random integration sites of the newly introduced genes and the influence of the chromatin structure and/or the regulatory elements at the sites of integration in the host genome [28]. One of the ways by which cancer cells facilitate gene expression is by overcoming the inhibitory effect of neighboring chromatin elements through interaction with MAR elements [29]–[31].

In this study, 4 out of the 5 identified MARs in the 682 kb DM in UACC-1598 cells caused a robust enhancement of gene expression while one showed a modest effect. This data support the notion that MARs can influence gene expression and that non-coding DM regions may contain functional MAR elements. Indeed, oncogene amplification alone may not be sufficient to induce high oncogene expression but may be achieved through co-amplification via functional MAR elements.

Recently, there have been reports indicating that MARs can organize and govern the accessibility to local chromatin structures and can indirectly influence transcription by insulating nearby genes [32]–[34]. Furthermore, DM MARs may influence the high order chromatin structure and can thus change the global expression of genes. In this regard, our data further suggest that the MAR elements in the 682 kb DM in UACC-1598 cells may play critical roles in oncogene activation in these cells.

## Supporting Information

Table S1 **Primers, annealing temperature, and size of predicted PCR products for scaffold/matrix DNA.** F and R indicate forward and reverse primers, respectively.
(DOC)

Table S2 **Primers, annealing temperature, and predicted PCR products for the construction of pGL3-promoter MARs vector.** F and R indicate forward and reverse primers, respectively.
(DOC)

Table S3 **Primers, annealing temperature, and size of PCR products for MYCN, EIF5A2 and beta-actin.** Primers MYCN-DNA, EIF5A2-DNA and Actin-DNA were for DNA amplification detecting; Primers MYCN-RNA, EIF5A2-RNA and Actin-RNA were for RNA transcription detecting. F and R indicate forward and reverse primers, respectively.
(DOC)
