# Supplementary material for: Novel Functional MAR Elements of Double Minute Chromosomes in Human Ovarian Cells Capable of Enhancing Gene Expression
Source: PLoS One. 2012 Feb 3;7(2):e30419. doi: 10.1371/journal.pone.0030419 (PMC3272018; doi:10.1371/journal.pone.0030419)
Supplement: Table S1 — Primers, annealing temperature, and size of predicted PCR products for scaffold/matrix DNA. F and R indicate forward and reverse primers, respectively. (DOC) [file pone.0030419.s001.doc]

Supplemental Table S1. Primers, annealing temperature, and size of predicted PCR products

for scaffold/matrix DNA.

| MARs | Primers | Annealing temperature | Size of PCR product (bps) |
| --- | --- | --- | --- |
| MAR1 | 5'-ATTCACCCTTGCCTTCCC-3'(F)  5'-TTGCCCATAGTCCCAGCT-3'(R) | 59 | 198 |
| MAR2 | 5'-GCCCTGTAGTCTGTTTGA-3'(F)  5'-CTTTATTAGGGTGGCAAA-3'(R) | 52 | 186 |
| MAR3 | 5'-TGAGATGGAGCCTTGTTT-3'(F)  5'-AATTAGCAGGGCGTGGTG-3'(R) | 56 | 152 |
| MAR4 | 5'-GGGACCAAGCATTCACTA-3'(F)  5'-ATTTACATTTCCCTCTGG-3'(R) | 54 | 120 |
| MAR5 | 5'-TGTAAACTCTATCACCCCACT-3'(F)  5'-ATATCACCTTAACCCAGTCAG-3'(R) | 59 | 957 |

F and R indicate forward and reverse primers, respectively.
