# Supplementary material for: Novel Functional MAR Elements of Double Minute Chromosomes in Human Ovarian Cells Capable of Enhancing Gene Expression
Source: PLoS One. 2012 Feb 3;7(2):e30419. doi: 10.1371/journal.pone.0030419 (PMC3272018; doi:10.1371/journal.pone.0030419)
Supplement: Table S2 — Primers, annealing temperature, and predicted PCR products for the construction of pGL3-promoter MARs vector. F and R indicate forward and reverse primers, respectively. (DOC) [file pone.0030419.s002.doc]

Supplemental Table S2. Primers, annealing temperature, and predicted PCR products for the construction of pGL3-promoter MARs vector

| MARS | Primers | Annealing temperature | Size of PCR products (bps) |
| --- | --- | --- | --- |
| MAR1 | 5'-GGGGTACCAGATCTATCTGGCAATTTCTCACT-3' (F)  5'-ACGCGTCGCCACTACACTCCACCCT-3' (R) | 56 | 565 |
| MAR2 | 5'-GGGGTACCAGATCTGTGTTTGAGTTCCTTGCT-3' (F)  5'-ACGCGTCGTTTATTGAATGGTCTTGG-3' (R) | 56 | 817 |
| MAR3 | 5'-GGGGTACCAGATCTGCTGCATTTATACTTGGG-3' (F)  5'-ACGCGTCGGTGATTCCTCTTTATATTGC-3' (R) | 56 | 567 |
| MAR4 | 5'-GGGGTACCAGATCTACAAAGAAACAAATCAGATGGG-3' (F)  5'-ACGCGTCGAACGTGGTGAAATCCTGTCTC-3' (R) | 61 | 625 |
| MAR5 | 5'-GGGGTACCAGATCTTGTAAACTCTATCACCCCACT-3' (F)  5'-ACGCGTCGATATCACCTTAACCCAGTCAG-3' (R) | 59 | 957 |

F and R indicate forward and reverse primers, respectively.
