# Supplementary material for: Novel Functional MAR Elements of Double Minute Chromosomes in Human Ovarian Cells Capable of Enhancing Gene Expression
Source: PLoS One. 2012 Feb 3;7(2):e30419. doi: 10.1371/journal.pone.0030419 (PMC3272018; doi:10.1371/journal.pone.0030419)
Supplement: Table S3 — Primers, annealing temperature, and size of PCR products for MYCN, EIF5A2 and beta-actin. Primers MYCN-DNA, EIF5A2-DNA and Actin-DNA were for DNA amplification detecting; Primers MYCN-RNA, EIF5A2-RNA and Actin-RNA were for RNA transcription detecting. F and R indicate forward and reverse primers, respectively. (DOC) [file pone.0030419.s003.doc]

Supplemental Table S3. Primers, annealing temperature, and size of PCR products for MYCN, EIF5A2 and beta-actin.

| Template | Primers | Annealing temperature | Size of PCR product (bps) |
| --- | --- | --- | --- |
| MYCN-DNA | 5'- ACCACAAGGCCCTCAGTAC -3'(F)  5'- GCAACGGCATTCTCTCAG -3'(R) | 57 | 233 |
| EIF5A2-DNA | 5'- TACTTGGCAGAGATTAAACAGG -3'(F)  5'- ACAAAGTATTTGCACCTTGAAG -3'(R) | 56 | 205 |
| Actin-DNA | 5'- ACCGCGAGAAGATGACCCAG -3'(F)  5'- TTAATGTCACGCACGATTTCCC -3'(R) | 57 | 295 |
| MYCN-RNA | 5'- TCCTCAAACGATGCCTTC -3'(F)  5'- ATGTTGTGGTTTCTGCGAC -3'(R) | 60 | 208 |
| EIF5A2-RNA | 5'- GTGGTGCTCAAAGGACGAC -3'(F)  5'- AGCAGGGAAAGGTAACCATC -3'(R) | 60 | 215 |
| Actin-RNA | 5'- CAGAAGGATTCCTATGTGG-3'(F)  5'- CATGATCTGGGTCATCTTC -3'(R) | 60 | 225 |

F and R indicate forward and reverse primers, respectively.
